# Electrons Surf Phason Waves in Moiré Bilayers

**DOI:** 10.1021/acs.nanolett.3c00490

**Published:** 2023-05-26

**Authors:** Indrajit Maity, Arash A. Mostofi, Johannes Lischner

**Affiliations:** Departments of Materials and Physics and the Thomas Young Centre for Theory and Simulation of Materials, Imperial College London, South Kensington Campus, London SW7 2AZ, U.K.

**Keywords:** phason, surfing, moiré materials, temperature-dependent properties, sliding, electrons

## Abstract

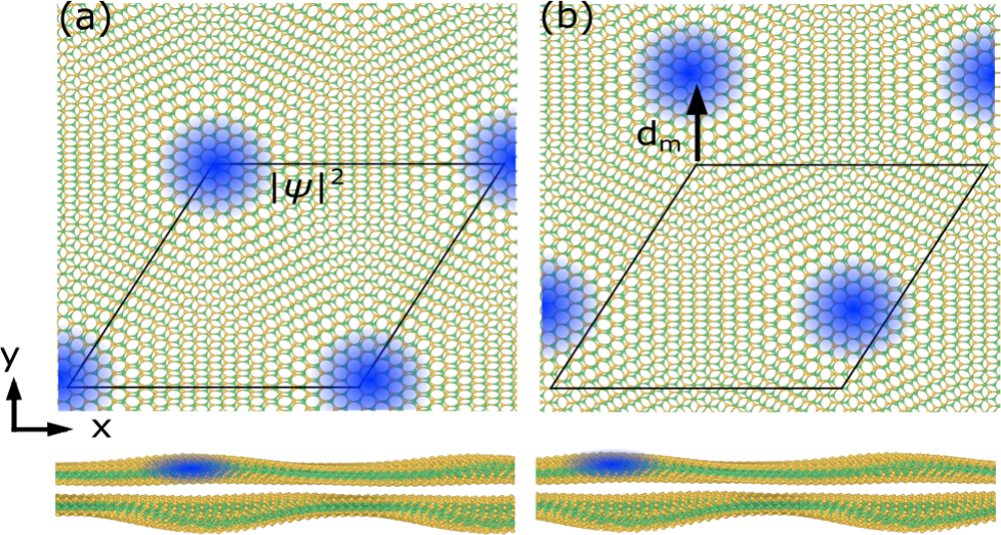

We investigate the effect of thermal fluctuations on the atomic and electronic structure of a twisted MoSe_2_/WSe_2_ heterobilayer using a combination of classical molecular dynamics and *ab initio* density functional theory calculations. Our calculations reveal that thermally excited phason modes give rise to an almost rigid motion of the moiré lattice. Electrons and holes in low-energy states are localized in specific stacking regions of the moiré unit cell and follow the thermal motion of these regions. In other words, charge carriers surf phason waves that are excited at finite temperatures. We also show that such surfing survives in the presence of a substrate and frozen potential. This effect has potential implications for the design of charge and exciton transport devices based on moiré materials.

Moiré materials, in which two or more two-dimensional (2D) materials are stacked and rotated relative to one another, have emerged as a new platform to discover, understand, and manipulate electronic properties, such as superconductivity and correlated insulating states.^1,2^ Twisting causes the electronic states to localize in specific regions of the moiré lattice, which results in a flattening of the corresponding bands and the emergence of strong electronic correlations. Because of this, moiré materials have been proposed as simulators of novel phases in quantum condensed matter.^3^

The effect of the twist on the electrons is often described in terms of an effective moiré potential which traps the electrons in specific regions of the moiré lattice. Recent experiments on twisted bilayer transition-metal dichalcogenides have estimated that the variation of this moiré potential across the moiré unit cell can be as large as 300 meV.^4^

To date, most theoretical studies have investigated the electronic structure of twisted multilayer systems at zero temperature and, hence, in a static moiré potential.^4−8^ However, temperature effects can be significant in moiré materials. This is because of so-called *moiré amplification*, whereby small atomic-scale thermal motion can give rise to large displacements of the moiré sites.^9^ At finite temperatures, therefore the moiré potential is dynamic, and the charge carriers reside in a highly mobile trapping potential. The behavior of a quantum particle in such a time-dependent trapping potential is an interesting problem^10−12^ with applications to quantum transients,^13^ transport of small particles using scanning tunneling microscopy or optical tweezers, and charge transport.^14^

In this Letter, we study the behavior of localized electrons and holes in twisted MoSe_2_/WSe_2_ heterobilayers at finite temperatures using a combination of classical molecular dynamics simulations and *ab initio* density-functional theory calculations. We find that the moiré sites are highly dynamic, which is a result of very soft phason modes that are thermally excited. Charge carriers follow the motion of the moiré sites to which they are localized and behave as though they are surfing on the dynamic moiré potential. The surfing speed is significantly different for twist angles close to 0° as compared to those close to 60°. We also discuss the impact of static frozen potential and a substrate on the surfing charge carriers.

Atomic structures of flat twisted MoSe_2_/WSe_2_ heterobilayers were generated using the TWISTER package.^15^ Because of the large size of the moiré unit cell, we used classical interatomic potentials fitted to *ab initio* density functional theory calculations to determine the relaxed atomic positions, study phonon properties, and carry out molecular dynamics simulations. Specifically, interactions between atoms in the same layer, i.e., intralayer interactions, were described using a Stillinger–Weber potential,^16^ while interlayer interactions were described using a Kolmogorov–Crespi potential.^17^ Structural relaxations and molecular dynamics simulations were performed using the LAMMPS package,^18^ and phonon calculations were performed using a modified version of the PHONOPY package.^19^ For the molecular dynamics simulations, a 3 × 3 moiré supercell was used. We have also performed simulations on larger supercells (up to 20 × 20) to investigate the dependence of our results on the system size.

Electronic structure calculations using *ab initio* density functional theory as implemented in the SIESTA package^20^ were performed on atomic structures obtained from the classical potentials. We included spin–orbit coupling^21^ in all our calculations. We used norm-conserving Troullier–Martins pseudopotentials^22^ and the local density approximation to describe exchange-correlation effects.^23^ Further details are provided in Section I of the Supporting Information.^24^

In Figures 1a and 1b we show the interlayer separation (ILS) landscape for relaxed twisted MoSe_2_/WSe_2_ bilayers with twist angles of 3.14° and 56.86°, respectively. For twist angles close to 0°, the moiré unit cell contains three high-symmetry stackings, which we label AA (Mo above W and Se above Se), B^Mo/Se^ (Bernal stacking with Mo above Se), and B^Se/W^ (Bernal stacking with Se above W). Among these, B^Mo/Se^ is energetically the most favorable. On the other hand, for twist angles close to 60°, the high-symmetry stackings in the moiré cell are AA′ (Mo above Se and Se above W), B^Mo/W^ (Bernal stacking with Mo above W), and B^Se/Se^ (Bernal stacking with Se above Se). Among these, AA′ is energetically the most favorable. In agreement with recent experiments,^4^ the calculated ILS landscape has a 6-fold symmetry around the AA stacking site for systems with twist angles near 0° and a 3-fold symmetry around the B^Se/Se^ site for systems with twist angles near 60°.

**Figure 1:**
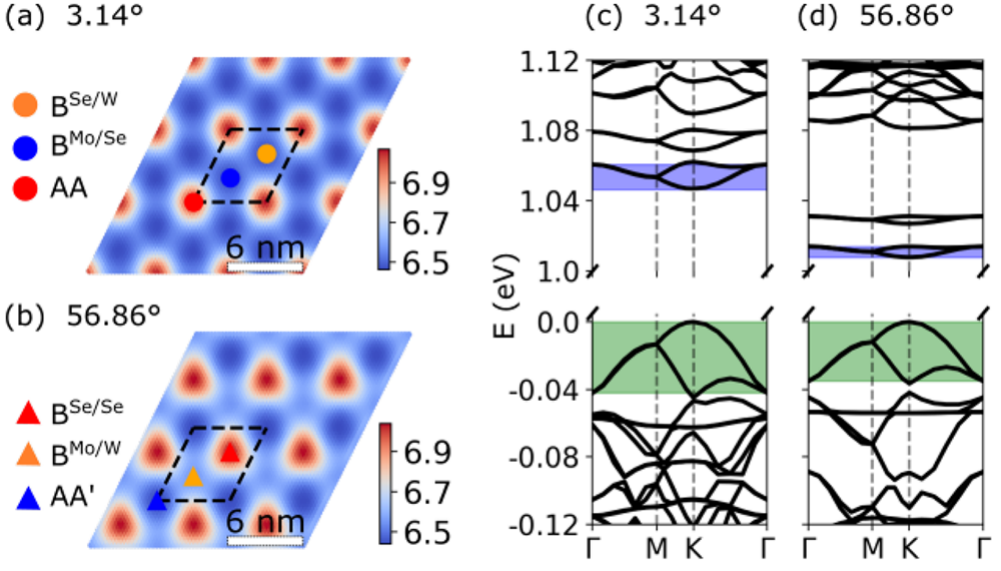
Interlayer separation (ILS) landscape for relaxed 3.14° (a) and 56.86° (b) twisted MoSe_2_/WSe_2_ heterobilayers. The color bar is in units of angstrom. The positions of high-symmetry stackings in the moiré unit cell (dashed line) are indicated by symbols. (c, d) Corresponding electronic band structures. The widths of the highest valence band and lowest conduction band are indicated by shaded areas. The zero of energy is set to the valence band maximum.

Figures 1c and 1d show the electronic band structures of relaxed 3.14° and 56.86° twisted heterobilayers, respectively. These systems form type II heterostructures with the valence band maximum (VBM) derived from the K valley of the WSe_2_ layer and the conduction band minimum (CBM) from the K valley of the MoSe_2_ layer. The widths of the VB and CB are smaller for twist angles close to 60°. For example, at 3.14°, the VB width is 42 meV and the CB width is 14 meV, while at 56.86° the VB with is 35 meV and the CB width is only 6 meV. To understand the origin of these differences, we also perform calculations on individual layers with the same atomic structure as in the relaxed twisted system (see Section II of the Supporting Information(24) for details). For the 56.86° system, the highest valence and the lowest conduction band from the corresponding monolayer calculations agree well with the twisted bilayer result. In contrast, this is not the case for the 3.14° system, indicating that interlayer tunneling plays a more important role for twist angles near 0° than for twist angles near 60°. Similar results have been reported for twisted homobilayers of transition-metal dichalcogenides.^25,26^ Homobilayers with twist angles close to 60° exhibit an inversion symmetry which—in combination with time-reversal symmetry—enforces the degeneracy of K-derived bands, indicating that off-diagonal interlayer tunneling terms vanish. In the corresponding heterobilayers, this inversion symmetry is broken because the metal atoms in both layers are different, but despite this, the interlayer tunneling is much weaker than in the systems with twist angles near 0°.

Figure 2 shows ILS landscapes of snapshots (separated by 0.1 ns) from the molecular dynamics simulations using a 3 × 3 supercell) at *T* = 150 K. It is observed that the moiré pattern stays largely intact but moves as a whole by several nanometers. Similar results are also found for smaller twist angles.^a^

**Figure 2:**
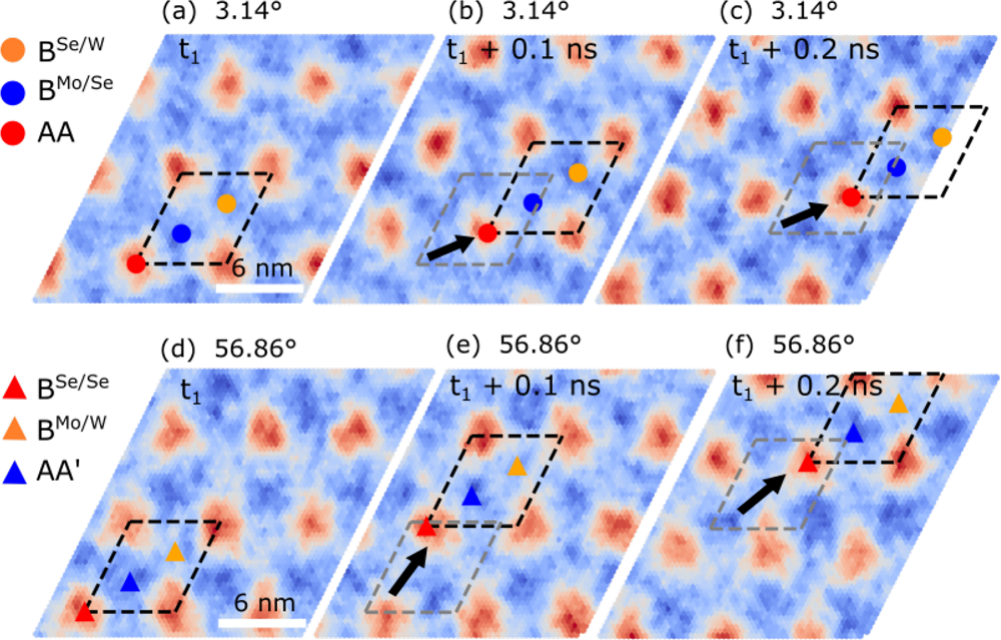
Time evolution of the interlayer separation landscape of twisted MoSe_2_/WSe_2_ heterobilayers during molecular dynamics simulations (using a 3 × 3 supercell) at *T* = 150 K, for twist angles of 3.14° (a–c) and 56.86° (d–f), with snapshots separated by 0.1 ns. The color bar is in units of angstroms. The positions of the high-symmetry stackings within the moiré unit cell are marked by symbols. The direction in which the moiré lattice moves is indicated by the black arrow, and the unit cell of the previous snapshot is marked with gray color.

We interpret the rigid motion of the moiré lattice at finite temperatures to be a consequence of thermally excited phason modes. A phason represents an effective global translation of the moiré lattice due to the uniform relative displacement of the two layers.^27,28^ b The energy cost associated with such modes is very small (<0.1 meV).^27−33^ Therefore, these modes are easily excited at finite temperatures. Moreover, our molecular dynamics simulations are nonperturbative and thus contain third- and higher-order anharmonic effects, which can be strong in twisted bilayers due to shallow sliding potential energy landscape (see the Supporting Information, Section VIII). We stress that by symmetry the system has no preferential direction for phason motion. In our simulations, however, we have found that the symmetry is lifted by the initial conditions. For example, the phasons move in different directions if the material is heated to 150 K by using different temperature increments (see the Supporting Information). In equilibrium, isotropy imposes that the thermal average of the phason velocity vanishes. However, this is no longer the case when a temperature gradient is created, for example, by heating a specific region of the sample. To demonstrate this, we have performed MD simulations in the presence of a temperature gradient. In such a system, the surfing motion occurs preferentially in the direction opposite to the applied temperature gradient. More details of the simulations are provided in the Supporting Information, Section X.

Figures 3a,b,g,h show the electronic band structures obtained for different molecular dynamics snapshots. The band structures are qualitatively very similar to those obtained for the relaxed atomic structure at *T* = 0 K. However, the electronic band gap is renormalized due to thermal fluctuations. In particular, we observe a band gap reduction of 37 ± 10 meV for 3.14° and 30 ± 8 meV for 56.86° systems at 150 K. Moreover, we find that the bandwidths of the top of the valence band and the bottom of the conduction bands are not sensitive to the temperature (see the Supporting Information, Section IX). Interestingly, we find that electrons and holes follow the movement of the moiré sites. For the 3.14° system, the VBM (CBM) always remains localized on the AA (B^Mo/Se^) site (see Figures 3e,f), while for the 56.86° system, the VBM remains localized on the AA′ site and the CBM on the B^Mo/W^ site (see Figures 3i–l). In other words, the electrons surf the phason waves that are excited at finite temperatures.

**Figure 3:**
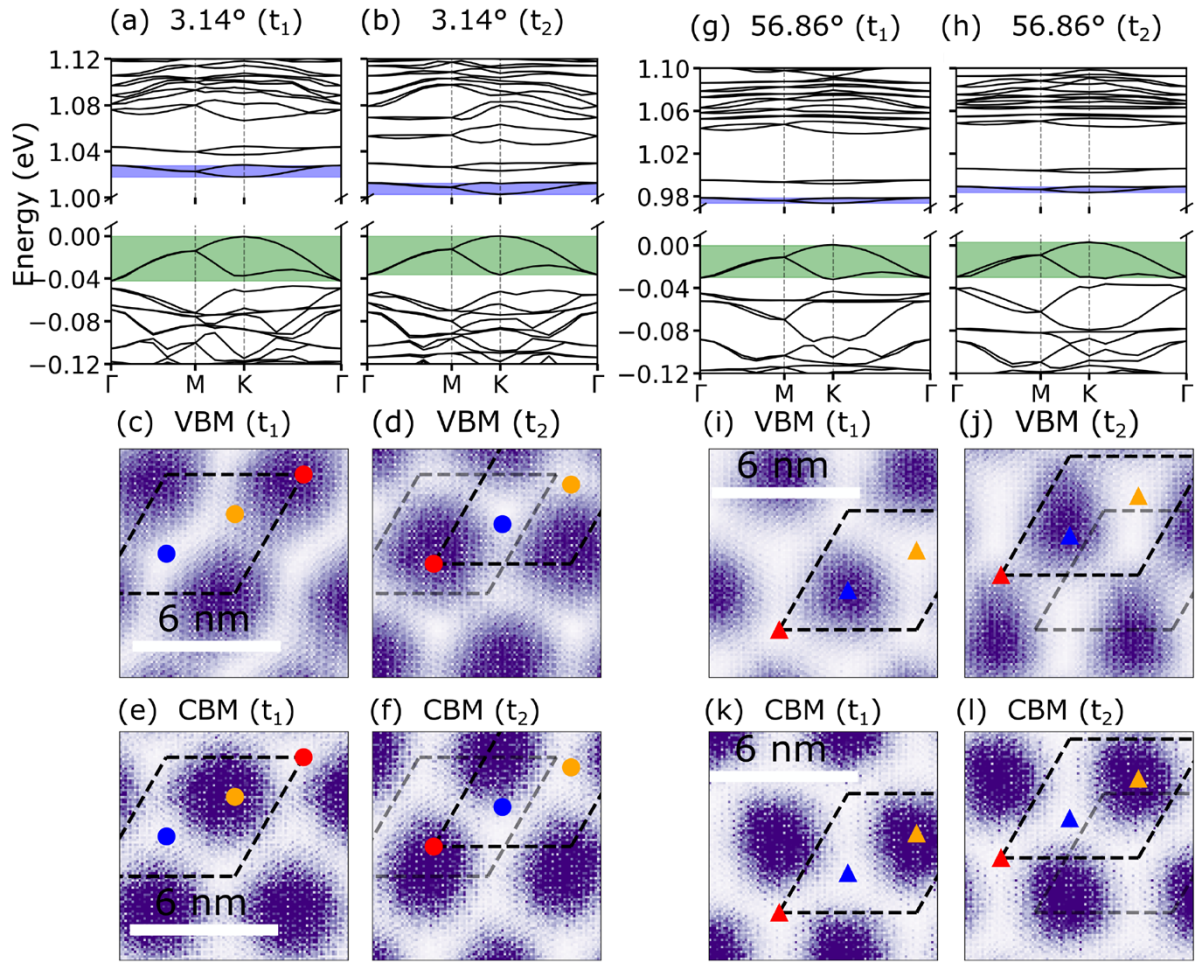
Top panels (a, b, g, h): electronic band structures for atomic structures obtained from molecular dynamics simulations at *T* = 150 K for a twisted MoSe_2_/WSe_2_ heterobilayer at two different times (*t*_1_ and *t*_2_ = *t*_1_ + 0.2 ns). Bottom panels (c, f, i, l): the squared absolute magnitudes of the VBM and CBM wave functions at the Γ-point of the moiré unit cell for the same atomic structure as in the top panels. The wave functions are averaged over the out-of-plane direction. The different high-symmetry stacking regions in the moiré unit cell are indicated by symbols.

We have also investigated the speed at which electrons surf. Interestingly, the moiré lattice amplifies atomic displacements, and therefore, small atomic displacements induced by thermal motion can give rise to significant displacements of the moiré sites. For instance, a displacement of 1 Å in the *x*-direction of all atoms of the MoSe_2_ layer in a 3.14° twisted heterobilayer gives rise to a displacement of the moiré sites by approximately 18 Å in the *y*-direction. The ratio of the moiré displacement and the atomic displacement is approximately , where *a*_m_, *a*, and θ are the moiré lattice constant, the lattice constant of MoSe_2_, and the twist angle, respectively. This is illustrated in Section IV of the Supporting Information.

To analyze the speed of surfing quantitatively, we define the mean distance traveled by a specific moiré site (e.g., AA site) as
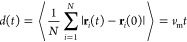
1where *N* is the total number of moiré unit cells (i.e., the number of moiré sites) in the supercell, **r**_*i*_(*t*) is the position of *i*th the moiré site at time *t*, ⟨···⟩ denotes the average over different molecular dynamics trajectories, and *v*_m_ is the surfing speed. We used 50 separate molecular dynamics trajectories with each trajectory containing 21 snapshots spanning over 0.1 ns. Interestingly, the electrons surf more slowly in systems with twist angles near 0° than in systems with twist angles near 60°. For example, we find *v*_m_ ≈ 25 m/s for 3.14° and *v*_m_ ≈ 40 m/s for 56.86° when a 3 × 3 supercell is used. For larger supercells, the surfing speed is reduced and approaches zero (see Figure S7). This can be understood from an analysis of the phonon dispersion relation (see Section VI of the Supporting Information): very close to the Γ point, the phasons exhibit a parabolic dispersion corresponding to a group velocity which is linear in crystal momentum.^c^ As the supercell size is increased in the MD simulations, smaller crystal momenta can be accessed, and therefore smaller surfing speeds are observed. In an experiment, the longest accessible wavevector is determined by the sample size. Typical samples of moiré materials have a linear extent of about 100 μm corresponding to a phason velocity of ≈5 mm/s (obtained by extrapolating our MD results). It is also important to note that at finite temperatures phasons with different wavevectors (and correspondingly different group velocities) can be thermally excited because the phason energy increases slowly as a function of wavevector near Γ.

We also compute the mean-square displacement of the moiré sites
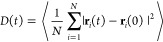
The mean-square displacement is found to be proportional to *t*^2^, indicating a free propagation of moiré sites (see Section V of the Supporting Information).^24^ In contrast, diffusive propagation would give rise to a mean-square displacement proportional to *t*. The free-propagating nature of surfing can be altered and become diffusive once the in-plane isotropy of the materials is broken. An example of such diffusive surfing is shown in the Supporting Information, Section X.

All results presented so far are for free-standing twisted heterobilayers. Experimental samples are typically placed on a substrate and contain frozen potential, for example, induced by extrinsic strain which can give rise to large twist-angle inhomogeneities.^4,35^ To study the effect of a substrate, we include a hexagonal boron nitride (hBN) substrate in our molecular dynamics simulations. We also observe the motion of the moiré sites and charge carrier surfing in these simulations, as shown in Figure 4 (blue, dashed line). Interestingly, the presence of a substrate increases the surfing speed: for a 3.14° twisted bilayer an increase of ∼40% is found when a 3 × 3 × 1 supercell is used.

**Figure 4:**
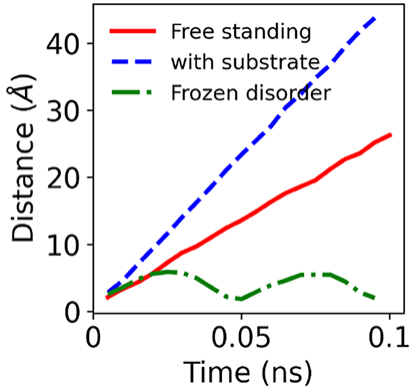
Distance traveled by surfing charge carriers as a function of time for (a) a free-standing twisted MoSe_2_/WSe_2_ bilayer, (b) a twisted MoSe_2_/WSe_2_ bilayer on a hexagonal boron nitride substrate, and (c) a twisted MoSe_2_/WSe_2_ bilayer in the presence of frozen potential. Results are obtained for a 3 × 3 supercell.

To study the impact of the frozen potential, we first relax the twisted bilayer on the substrate and then perform a molecular dynamics simulation in which the substrate atoms are not allowed to move. Then, the substrate acts as a frozen potential on the atoms in the twisted bilayer. In these simulations, we do not observe free propagation of moiré sites (Figure 4, green, dot-dashed line). This is a consequence of frozen potential induced pinning of phasons. Such pinning was also found in twisted bilayer graphene in a recent theoretical study.^36^ Even though there is no free propagation of moiré sites, there are large displacements of ∼5 Å of the sites around their equilibrium position resulting from the moiré amplification of thermal fluctuations.

The frozen potential-induced pinning can be overcome by increasing the temperature. In order to demonstrate this, we perform molecular dynamics simulations at *T* = 1200 K in the presence of the frozen potential and observe again the free propagation of moiré sites (see the Supporting Information, Section VIII). Significant movement of moiré sites due to finite temperature in twisted bilayer graphene was observed in a recent experiment.^37^ Similarly, the free sliding of twisted bilayer graphene nanoflakes due to thermal fluctuations has been reported.^38^ This clearly indicates that high-quality twisted bilayer materials are key to observing the surfing motion.

Another way to detect and exploit charge carrier surfing is through sliding one layer of the twisted heterobilayer slides relative to the other layer. This generates a motion of the moiré sites in the transverse direction, as shown in Section IV of the Supporting Information. As the electrons follow the motion of the moiré sites, a transverse current is generated. Specifically, if the MoSe_2_ layer is pulled along the *x*-direction with a speed *v*^*x*^, the moiré sites move along *y*-direction with a speed . The current density that is generated by the electron surfing is given by , where *n* is the charge density and *e* is the electron charge. Electron transport in such a chiral moiré charge pump could be topological as discussed in a related context in twisted bilayer graphene using continuum models and tight-binding models.^39−41^

We have demonstrated that the moiré sites of a twisted MoSe_2_/WSe_2_ heterobilayer move at finite temperatures due to thermally excited phasons. Electrons and holes follow the motion of the moiré sites and thus surf the phason waves. We have also discussed the impact of the substrate and frozen potential on free surfing. Our findings are relevant for the design of transport devices based on moiré materials.

## Supporting Information Available

More details of the calculations, the electronic band structure with isolated atomic reconstructed monolayer MoSe_2_ and WSe_2_, the phason eigenvectors, illustration of moiré amplification, moiré site movements, the phonon dispersions, system size dependence of the surfing speed, and temperature-dependent band structures (PDF)
Movie S1 (MP4)
Movie S2 (MP4)
Movie S3 (MP4)
Movie S4 (MP4)
Movie S5 (MP4)

## Footnotes

a However, no such movement of the moiré lattice is observed for untwisted bilayers, i.e., at twist angles of 0° and 60°.

b Note that the actual polarization vectors often display a more complicated spatial structure; see Section III of the Supporting Information.^24^

c The phason dispersion is linear to arbitrarily small momenta only in a truly incommensurate system.^34^
