# Supplementary material for: Electrons Surf Phason Waves in Moiré Bilayers
Source: Nano Lett. 2023 May 26;23(11):4870–5. doi: 10.1021/acs.nanolett.3c00490 (PMC10273461; doi:10.1021/acs.nanolett.3c00490)
Supplement: Supplementary file 1 — nl3c00490_si_006.pdf [file nl3c00490_si_006.pdf]

Supplementary Information (SI) :
Electrons surf phason waves in moiré bilayers

Indrajit Maity, Arash A. Mostofi,* and Johannes Lischner†

Departments of Materials and Physics and the Thomas Young Centre for Theory and Simulation of Materials, Imperial College London, South Kensington Campus, London SW7 2AZ, UK

I: SIMULATION DETAILS

Generation of structures

All the structures for the following twist angles were generated by the TWISTER package [S1]. The unit-cell lattice constants for both the $MoSe_2$ and WSe_2 layer were set to 3.32 Å while generating the moiré patterns.

Twist angles	Number of atoms	Moiré length (in Å)
3.14°	1986	60.4
56.86°	1986	60.4

TABLE I: Moiré patterns of twisted bilayer of $MoSe_2/WSe_2$ studied in this work.

Structural relaxations

The moiré patterns are relaxed using the LAMMPS package with the Stillinger-Weber [S2], and Kolmogorov-Crespi [S3] potentials to capture the intralayer and interlayer interactions of the twisted bilayer of WSe_2, respectively. The Kolmogorov-Crespi parameters used in this work can correctly reproduce the interlayer binding energy landscape, obtained using density functional theory. The atomic relaxations produced using these parameters are in excellent agreement with relaxations performed using density functional theory. We relax the atoms within a fixed simulation box with the force tolerance of 10^{-5} eV/Å for any atom along any direction.

Molecular dynamics simulations

We equilibrate the moiré material under periodic boundary conditions in the canonical ensemble at several temperatures for a nano-second using a Noseé-Hoover thermostat. We track the dynamics of moié sites using the micro-canonical ensemble for several nanoseconds. To extract the speed of the moiré sites, we have used a $3 \times 3 \times 1$ moiré supercell. Additional molecular dynamics simulations were performed for smaller twist angles (1° and 59°). Similar movements of moiré sites were observed. To simulate different initial conditions, we used a

Langevin thermostat with different increments in temperature within a loop up to 150 K before equilibration. The surfing speeds for all these different initial conditions are obtained for 3.14° moiré $3 \times 3 \times 1$ supercell to be 31, 40, 16 nm/ns.

Inclusion of the substrate and frozen potential

We use hexagonal Boron Nitride (hBN) as a substrate for the $MoSe_2/WSe_2$ twisted bilayer. The intralayer interactions were described using a Tersoff potential [S4] and the interlayer interactions were captured using a Kolmogorov-Crespi potential [S5]. The lattice constant for hBN was 2.516 Å and 24×24 unit cells were added to the twisted bilayer. As long as the dynamics of the hBN and the twisted bilayer are allowed at finite T, we find the moiré sites move. However, if we freeze the movements of hBN in all directions, the moiré sites stop free movement but show large displacements around a mean position. The frozen hBN atoms in such a scenario act as a frozen potential which pin the phason motion.

Electronic structure calculations

We use a double-ζ plus polarization basis for the expansion of wavefunctions. For all the electronic structure calculations we use the Γ point in the moiré Brillouin zone to obtain the converged ground state charge density. A large vacuum spacing of 20 Å is used in the out-of-plane direction for all the density functional theory (DFT) calculations. All the electronic structure calculations at finite temperature are performed using a snapshot of the moiré unit cell from the classical molecular dynamics simulations. For computing the averaged electronic band gap at 150 K, we used 6 snapshots from our molecular dynamics simulations. Note that all the electronic structure calculations were performed on the moiré unit cell (i.e., $1 \times 1 \times 1$ moiré supercell).

II : ELECTRONIC BAND-STRUCTURE OF 3.14° AND 56.86° TWISTED $MOSE_2/WSE_2$

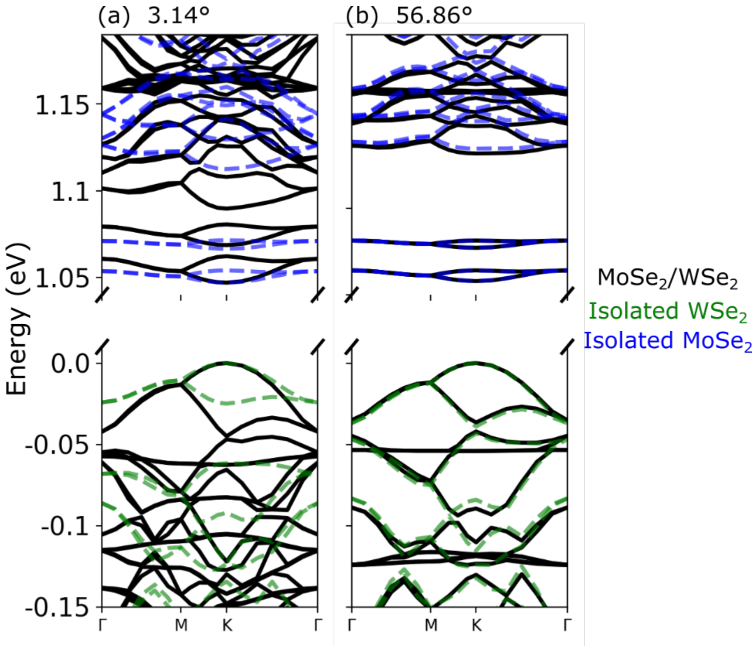

FIG. S1: Electronic band-structure calculations of 3.14° and 56.86° twisted $MoSe_2/WSe_2$ heterobilayer. The band-structure calculations are compared with monolayer $MoSe_2$ and WSe_2 after isolating from the relaxed twisted bilayer calculations. No further relaxations on the isolated monolayers are performed. The valence band maximum of the twisted bilayer has been set to 0 eV. For better comparison, the valence band maximum of WSe_2 and the conduction band minimum of the $MoSe_2$ are aligned to those of twisted heterobilayer calculations.

III : PHASONS OF 3.14° TWISTED MOSE$_2$/WSE$_2$

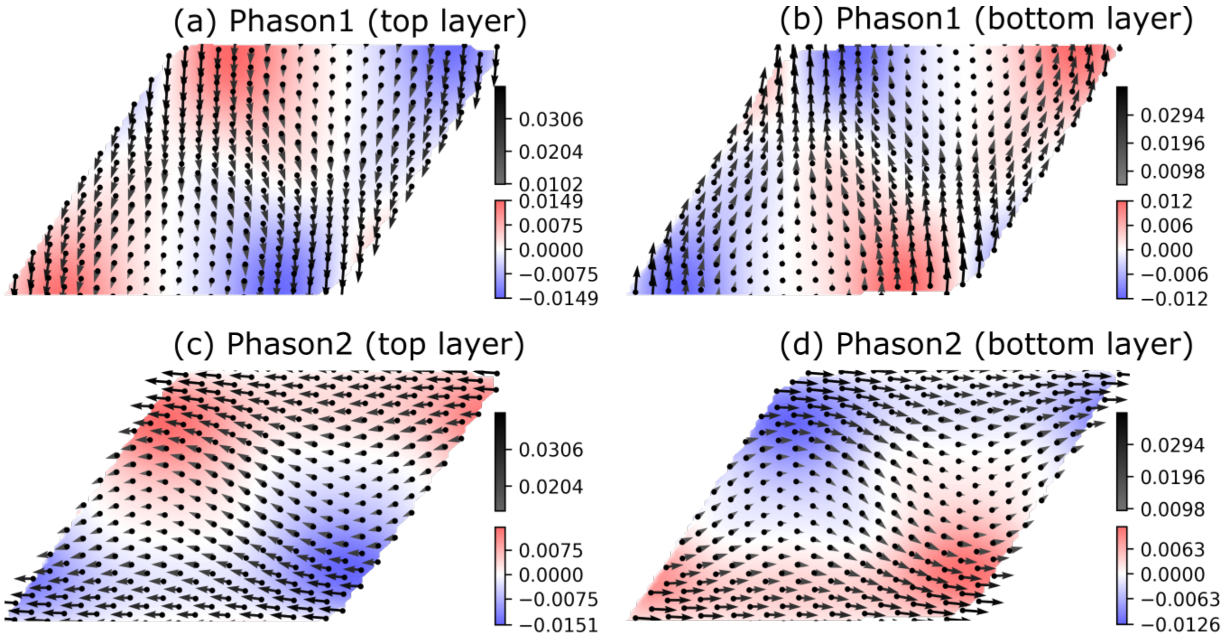

FIG. S2: Polarization vectors associated with the ultra-low frequency phason modes with energy 0.07 meV at the Γ point shown for a moiré unit cell of the twisted $MoSe_2/WSe_2$ heterobilayer. The arrows (gray colorbar) denote in-plane displacements (only for Mo atoms of the top layer and W atoms for the bottom layer), whereas out-of-plane displacements are represented as a continuous field (colored). The origin of the arrow indicates the position of a metal atom.

IV: ILLUSTRATION OF MOIRÉ AMPLIFICATION

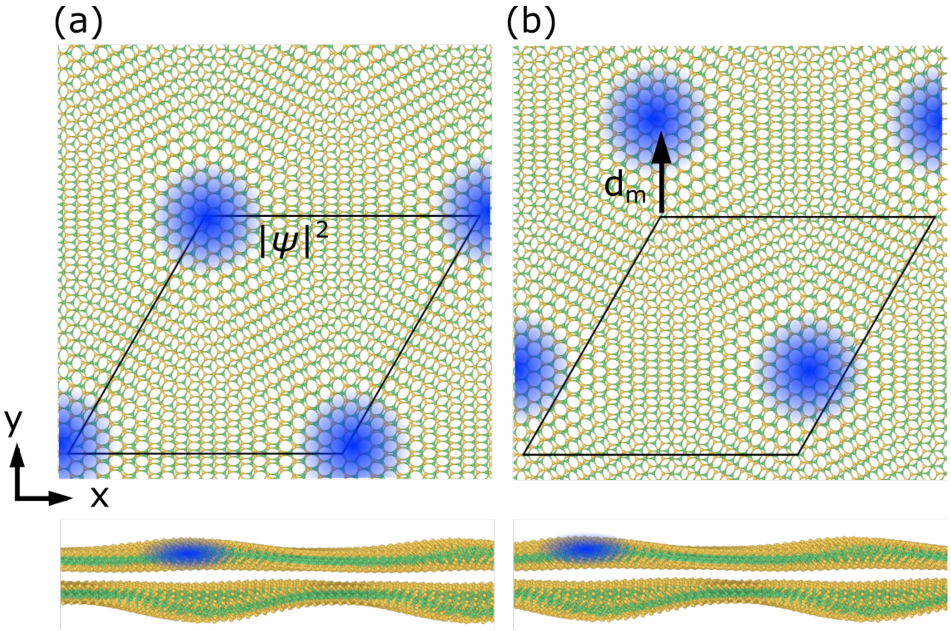

FIG. S3: Schematic of the moiré amplification of atomic displacements and the associated transport or "surfing" of charge carriers in a 3.14° twisted $MoSe_2/WSe_2$ bilayer. (a): Initial atomic structure and squared magnitude $|\psi|^2$ of the VBM wavefunction at the Γ-point of the moiré Brillouin zone. The moiré unit cell is indicated. (b) Atomic structure after the atoms in the top layer have been displaced by 1 Å along x-direction. The AA site where the VBM is localized moves by $d_m \approx 18$ Å along the y-direction. The top panels show the view from the top and the bottom panels show the view from the side. A moiré unit cell is indicated at a fixed position in both figures (solid black line). The out-of-plane displacements of the atoms are exaggerated for visual clarity in the bottom panels.

V : MOTION OF MOIRÉ SITES

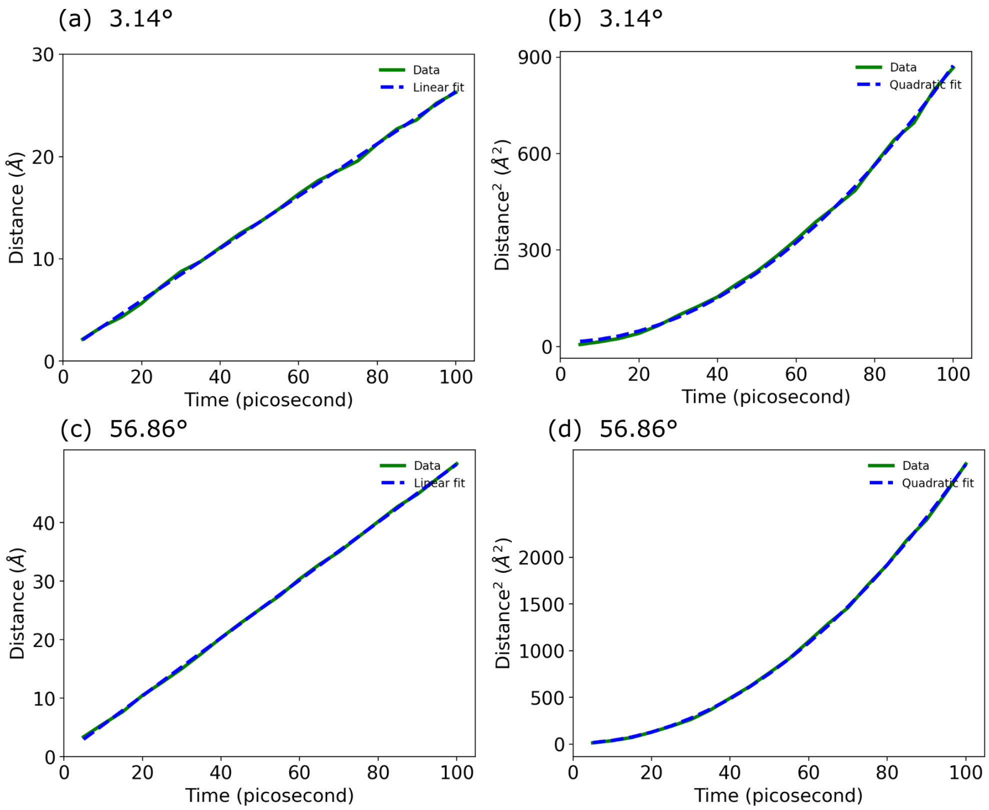

FIG. S4: Distance and mean-square distance vs. time computed for $MoSe_2/WSe_2$ for 3.14° and 56.86° twist angles.

VI : PHONON DISPERSION OF 3.14° TWISTED $MOSE_2/WSE_2$

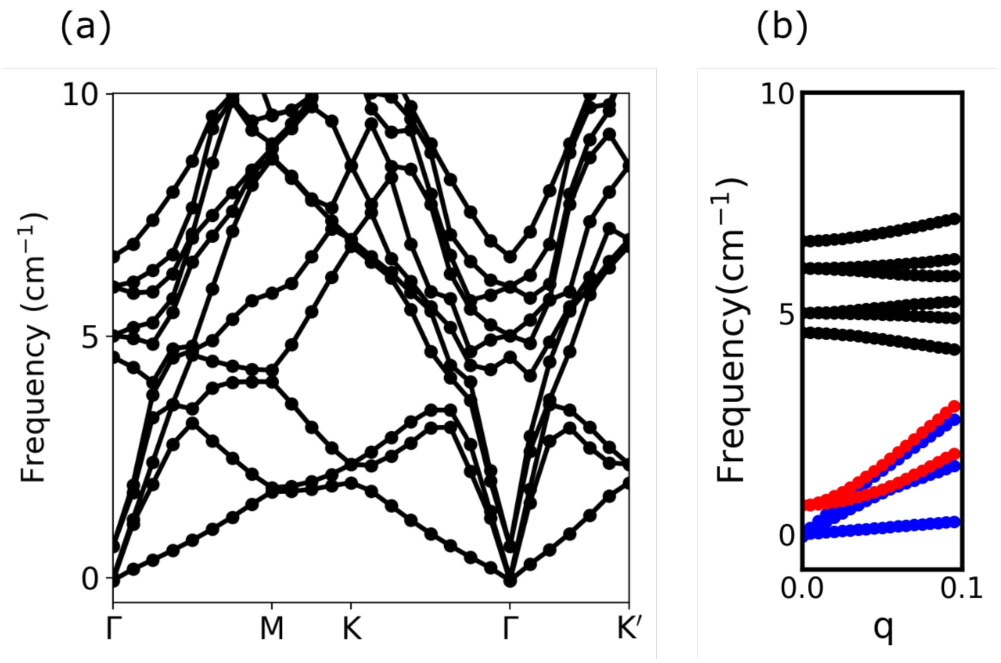

FIG. S5: (a) Phonon dispersion of the 3.14° twisted $MoSe_2/WSe_2$ calculated at T=0 K. (b) Phonon dispersion for very close to the Γ point along the $\Gamma - K$ direction (in crystal coordinates). The conventional acoustic modes are highlighted with blue colour. On the other hand, the phason modes are highlighted with red colour. We also extract the group velocity by fitting a line to the linearly dispersing phason modes. The phason velocities are 7.8×10^2 m/s and 3.4×10^2 m/s, respectively. The group velocities associated with the phason modes significantly decrease very close to the Γ point as can be seen by deviation from the straight line behaviour. This is in sharp contrast to the acoustic modes (LA/TA modes), where the group velocity remains constant up to arbitrarily small momentum.

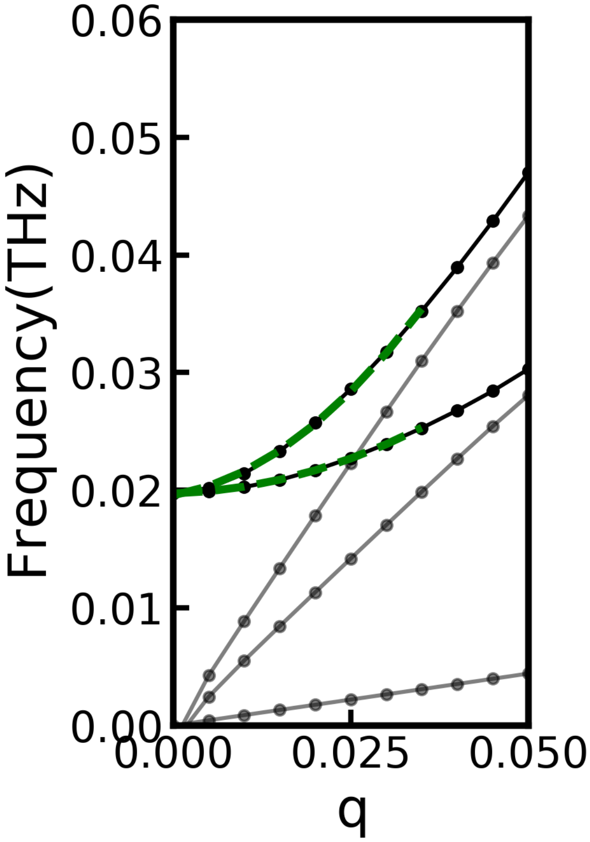

FIG. S6: Phonon dispersion for the 3.14° twisted $MoSe_2/WSe_2$ heterobilayer with the phason dispersion fitted with a quadratic function. The fitted data are shown with green dashed lines.

VII: SIMULATION CELL SIZE DEPENDENCE OF THE SURFING SPEED FOR 3.14° TWISTED $MoSe_2/WSe_2$

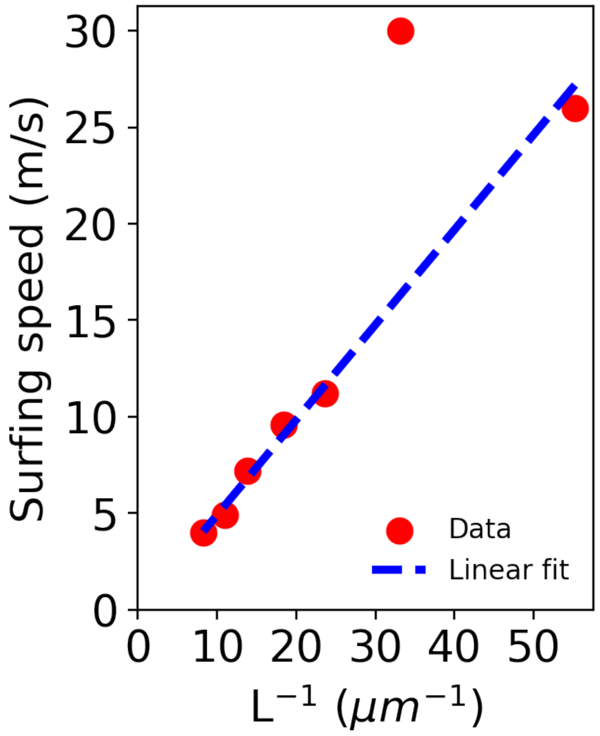

FIG. S7: The surfing speed is linearly dependent on the inverse of sample size. Using the linear fit, we have extracted the surfing speed for experimentally relevant samples.

VIII: SURFING MOVIES

Shallow Potential Energy Landscape

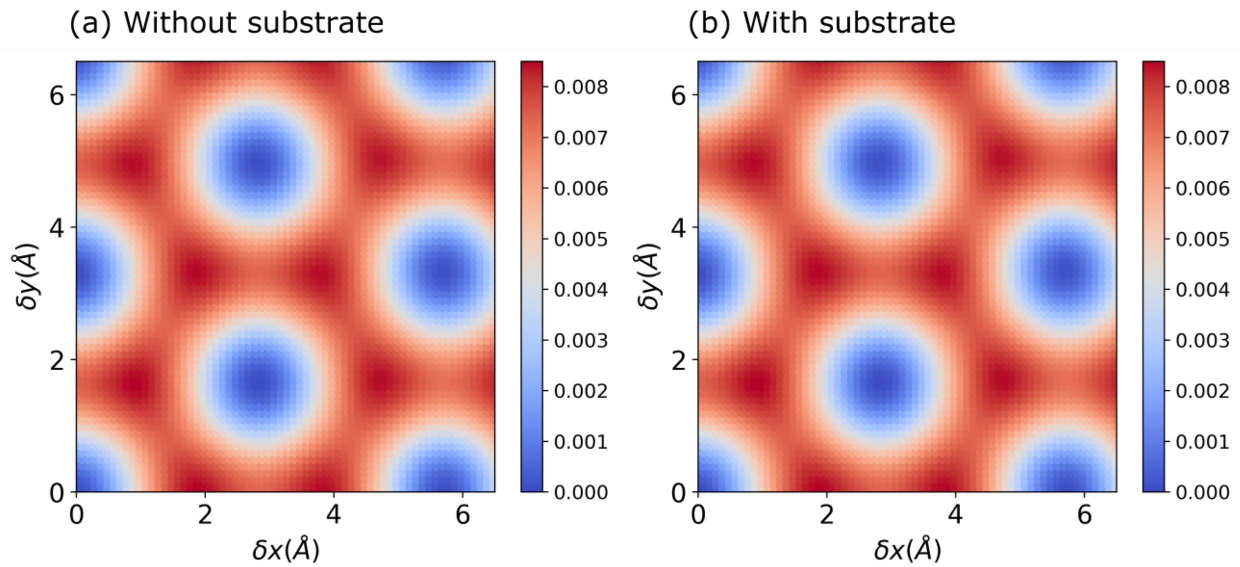

FIG. S8: (a),(b): The sliding potential energy surfaces for 3.14° twisted $MoSe_2/WSe_2$ heterobilayer without and with the inclusion of the hBN substrate. The colorbar represents energy/atom with the units of eV/atom.

Frozen potential

The attached video (*Surfingindisorder.mp4*) shows the atomic motion (obtained from a molecular dynamics simulation) in the presence of frozen potential as described in the main text at a high temperature of 1200 K. We find that surfing survives even in the presence of frozen potential. Also, we present the same dynamics (*Surfingindisordersideview.mp4*) from a side view focusing on the atomistic details to emphasize the moiré magnification that happens at the moiré scale.

Long time scale with different initial conditions

The movements of the moiré sites over a large time window for three different initial conditions are presented as movies *Movementlargewindow1.mp4*, *Movementlargewindow2.mp4*,

Movementlargewindow3.mp4. Each movie was created with 8 ns long trajectories. Moreover, the direction changes when sufficiently long simulations are carried out (here we use a simulation time of 8 ns while in the original manuscript, the simulation time was less than 1 ns). The situation is similar to a drunkard's walk where the drunkard might initially take a couple of steps in a specific direction. In such a situation, even though there's no preferred direction for the steps taken, after n steps the walker traverses a distance proportional to $\sqrt{n}$.

IX: TEMPERATURE-DEPENDENT ELECTRONIC BAND STRUCTURES

We have computed the bandwidths of the top of the valence band and the bottom of the conduction bands and found that the bandwidths are insensitive to temperature. These results are summarized in table II. Also, we show the electronic bandstructure plots by averaging the results from five snapshots of our molecular dynamics simulations. These results are presented in Fig. S9.

	Bandwidth in meV			
	$T = 0$ K		$T = 150$ K	
Twist angle	V1	C1	V1	C1
3.14°	42	14	39	11
56.86°	35	6	32	6

TABLE II: Comparison of bandwidths in the twisted bilayers at T=0 K and 150 K for the top of the valence band V1 and the bottom of the conduction band C1.

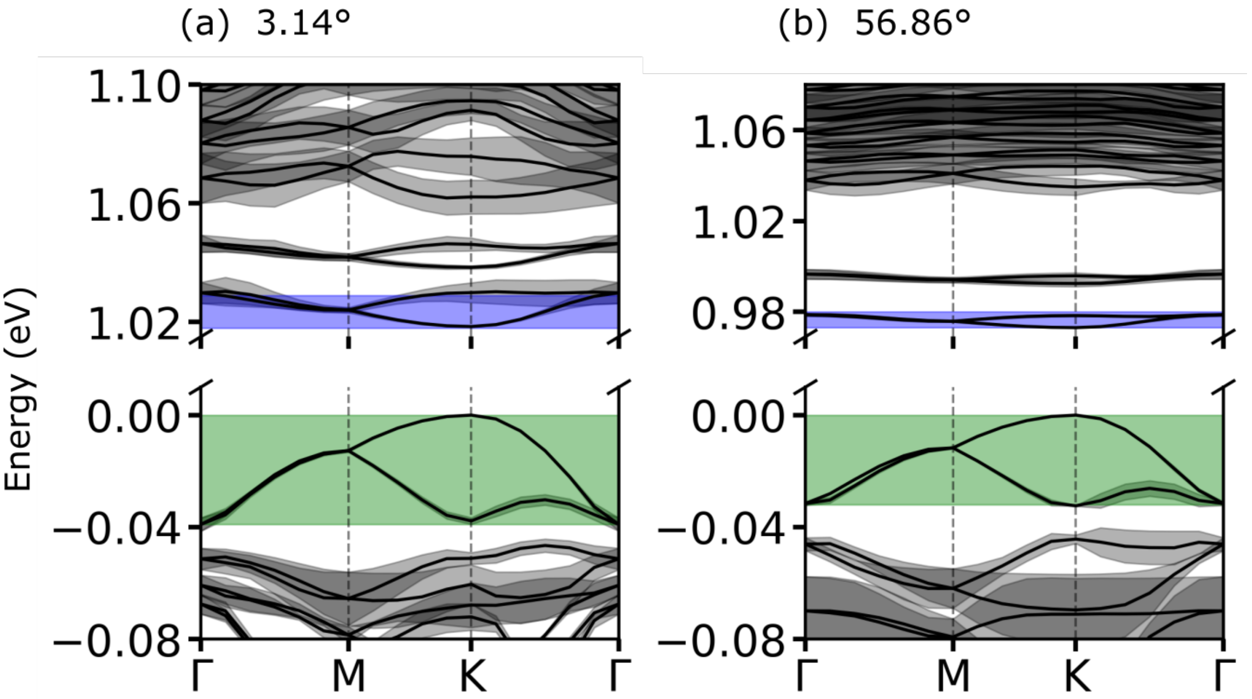

FIG. S9: Averaged electronic band structure of twisted $MoSe_2/WSe_2$ heterobilayer for 3.14° and 56.86° twist angles at T=150 K. The bandwidths of the valence band (green color) and the conduction band (blue color), and the standard deviations of the bands are shaded with grey color. Note that in all our calculations the top of the valence band is set to zero and the bottom of the conduction band is set to a constant. This is done in order to separate the band gap fluctuations and the bandwidth fluctuations. The bandgap fluctuations are reported in the main text.

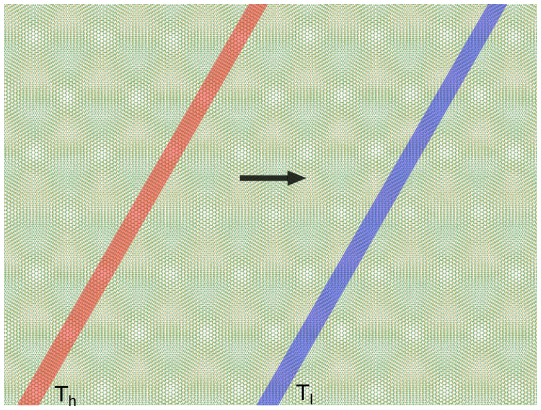

FIG. S10: (a) Moiré supercell with an applied temperature gradient. The temperature of the red region is set to T_h and the temperature of the blue region is set to T_l with $T_h > T_l$. The surfing direction is marked with an arrow.

X: TEMPERATURE-GRADIENT TO BREAK ISOTROPY

To demonstrate this, we have created a temperature gradient as shown in Figure S10. We have computed the mean-square-displacements of the moiré sites with a $3 \times 3 \times 1$ and a $7 \times 7 \times 1$ moiré supercell with $T_h = 175$ K and $T_l = 125$ K and found it to be linear in time, indicating diffusive motion. We have used Langevin thermostats to maintain the temperature in hot and cold regions [S6]. The average temperature of the system is kept at 150 K.

* a.mostofi@imperial.ac.uk

† j.lischner@imperial.ac.uk

[S1] S. Naik, M. H. Naik, I. Maity, and M. Jain, Computer Physics Communications **271**, 108184 (2022).

[S2] J.-W. Jiang and Y.-P. Zhou, *Handbook of Stillinger-Weber Potential Parameters for Two-Dimensional Atomic Crystals* (IntechOpen, Rijeka, 2017).

[S3] M. H. Naik, I. Maity, P. K. Maiti, and M. Jain, The Journal of Physical Chemistry C **123**, 9770 (2019).

[S4] J. Tersoff, Phys. Rev. B **38**, 9902 (1988).

[S5] H. Li, S. Li, M. H. Naik, J. Xie, X. Li, J. Wang, E. Regan, D. Wang, W. Zhao, S. Zhao, S. Kahn, K. Yumigeta, M. Blei, T. Taniguchi, K. Watanabe, S. Tongay, A. Zettl, S. G. Louie, F. Wang, and M. F. Crommie, Nature Materials **20**, 945 (2021).

[S6] T. Ikeshoji and B. Hafskjold, Molecular Physics **81**, 251 (1994).
